# Sex influences the effect of adiposity on arterial stiffness and renin‐angiotensin aldosterone system activity in young adults

**DOI:** 10.1002/edm2.317

**Published:** 2021-12-26

**Authors:** Cindy Z. Kalenga, Sharanya Ramesh, Sandra M. Dumanski, Jennifer M. MacRae, Kara Nerenberg, Amy Metcalfe, Darlene Y. Sola, Sofia B. Ahmed

**Affiliations:** ^1^ Cumming School of Medicine University of Calgary Calgary Alberta Canada; ^2^ Libin Cardiovascular Institute University of Calgary Calgary Alberta Canada; ^3^ Temerty Faculty of Medicine University of Toronto Toronto Ontario Canada; ^4^ Alberta Kidney Disease Network Calgary Alberta Canada; ^5^ O’Brien Institute for Public Health University of Calgary Calgary Alberta Canada; ^6^ Alberta Children's Hospital Research Institute Calgary Alberta Canada

**Keywords:** adiposity, arterial stiffness, body mass index, female, male, pulse wave velocity, renin angiotensin aldosterone system, sex

## Abstract

**Introduction:**

Sex influences the cardiovascular risk associated with body mass index (BMI) in older adults. Whether this risk differs by sex in younger adults is unknown. We aimed to evaluate the association between measures of adiposity and arterial stiffness and reninangiotensin‐aldosterone system (RAAS) activity in younger adults, stratified by sex.

**Methods:**

Body mass index (BMI), waist circumference (WC), hip circumference (HC), waist‐to‐hip ratio (WHR), waist‐to‐height ratio (WHtR) and fat mass% (FM%) were measured in healthy participants in a fasting, high‐salt state. Arterial stiffness [pulse wave velocity (PWV) and aortic augmentation index (AIx)] were measured at baseline and in response to angiotensin II challenge, a validated marker of RAAS activity. Associations were evaluated using linear regression analysis and stratified by sex.

**Results:**

Ninety‐five healthy, normotensive, non‐diabetic females (*n* = 67, 37 ± 2 y, BMI 25 ± 1 kg/m^2^) and males (*n* = 28, 39 ± 3 y, BMI 27 ± 1 kg/m^2^) participated in the study. No association was observed between any measure of adiposity and PWV, either at baseline or in response to angiotensin II challenge in premenopausal females. In contrast, all measures of adiposity except HC were associated with PWV at baseline (BMI *r* = 0.32; WC *r* = 0.18; WHtR *r* = 0.34; FM *r* = 0.21; all values *p* < .05) and in response to AngII (BMI *r* = −0.39; WC *r* = −0.42; WHR *r* = −0.39; and WHtR *r* = −0.55) in males. Most adiposity measures were positively associated with baseline AIx (BMI *r* = 0.33; WC *r* = 0.27; WHtR *r* = 0.35; FM% *r* = 0.25; *p* < .05) in females, while only WHtR was associated with baseline AIx (*r* = 0.39; *p* = .04) in males. All measures of adiposity were positively associated with a blunted Aix response to Ang II (all values *p* < .001) in females. BMI, WC, WHR and WHtR were associated with a blunted AIx response to Ang II (ΔAIx: BMI *r* = −0.37; WC *r* = −0.31; WHR *r* = −0.16; and WHtR *r* = −0.22; all values *p* < .05) in males.

**Conclusion:**

The associations between adiposity measures and cardiovascular risk differ by sex in a young population. These factors should be considered when managing cardiovascular risk.

## Graphical abstract

The optimal sex‐specific measure of adiposity in young adult population is unclear. We evaluated the association between measures of adiposity, arterial stiffness and renin‐angiotensin‐aldosterone system (RAAS) activity in younger adults, stratified by sex. Our study showed that associations between adiposity measures and cardiovascular risk differ by sex in a young population.

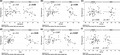

## 1 INTRODUCTION

Obesity is a global epidemic,^1^, ^2^ and cardiovascular disease is the leading cause of death worldwide.^3^ Body mass index (BMI), an important modifiable cardiovascular risk factor,^4^ is increasing at a greater rate in women compared to men,^5^ which may contribute to the stagnation in changes in incidence and mortality of coronary heart disease in younger people, and specifically younger women.^6^, ^7^ However, studies^8^, ^9^, ^10^, ^11^, ^12^ suggest that adiposity measures other than BMI may provide a more accurate sex‐specific estimate of cardiovascular risk in older adulthood.^13^, ^14^ While obesity in young adulthood is associated with future cardiovascular risk,^15^ the optimal sex‐specific measure of adiposity in this population is unclear. Furthermore, BMI does not differentiate between type of adipose tissue or fat distribution which differ by sex^16^, ^17^, ^18^ and both have been shown to affect cardiovascular risk.^16^, ^19^ Moreover, visceral fat, which is located primarily around the abdomen, as compared to subcutaneous fat, which is primarily located around the hips and thighs, is associated with increased renin‐angiotensin‐aldosterone system activity (RAAS),^19^ upregulation of which is deleterious to cardiovascular outcomes.^20^, ^21^, ^22^ A study of women and men >50 years of age showed sex differences in how fat distribution influenced the association between BMI and arterial stiffness, a validated marker of cardiovascular risk,^19^ though whether these results are applicable to the young, healthy population is unknown.^8^

We have previously shown that sex modifies the effect of BMI on the blood pressure and renal haemodynamic response to angiotensin II,^23^ a validated marker of RAAS activity.^24^ We sought to determine the effect of sex on BMI and other measures of adiposity on arterial stiffness and arterial RAAS activity in young, healthy females and males. We hypothesized that the association between BMI and arterial stiffness and RAAS activity would differ by sex, and measures of visceral adiposity would be positively associated with arterial RAAS activity.

## 2 METHODS

### 2.1 Participants

Ninety‐five healthy (67 females, 28 males), normotensive, non‐diabetic, non‐smoking participants were enrolled in the study. Participants underwent a medical history, physical examination and laboratory screening. The study protocol was approved by the Conjoint Health Research Ethics Board at the University of Calgary. Written informed consent was obtained from all study participants in accordance with the Declaration of Helsinki.

### 2.2 Protocol

Participants were instructed to consume >200 mmol sodium day for 3 days before the study to ensure maximal RAAS suppression.^25^ All participants provided a second morning spot urine for determination of urinary sodium to verify compliance with the high‐salt diet.^26^ Participants were studied in the supine position in a warm, quiet room after an 8 h fast. All females were studied 14 days after the 1st day of the last menstrual period, determined by counting days and measuring 17β‐estradiol levels. At 8 a.m., an 18‐gauge peripheral venous cannula was inserted into the antecubital vein of each arm (1 for infusion, 1 for blood sampling). After a 90‐min equilibration period, arterial stiffness was measured at baseline and after 60 min in response to two doses of Ang‐II (3 ng × kg^−1^ × min^−1^ × 30 min, 6 ng × kg^−1^ × min^−1^ × 30 min) as an index of RAAS activity.^27^ Blood samples were collected at baseline and every 30 min until the end of the study. BP was recorded every 15 min by an automatic recording device (Dinamap; Critikon, Tampa, FL). Participants were studied in the supine position using a standard cuff placed on the right arm at rest. The mean of two readings taken by the same registered nurse was reported.

### 2.3 Measurements of adiposity

Weight was measured via digital scale and standing height with a tape measure. BMI was calculated as weight/(height)^2^. Standing waist circumference (WC) was measured between the last rib and the iliac crest. Hip circumference (HC) was measured at the maximum buttock circumference. Fat mass (FM) was evaluated by bioelectrical impedance (RJL Sciences QuantumII). Resistance (R) and reactance (Xc) were measured with the RJL Systems, Model Quantum II, a four‐terminal single‐frequency (800 μA at 50 Khz) impedance plethysmograph (RJL Systems, Detroit, MI) with an internal calibration system. Participants wore light clothing and were barefoot. The participant reclined in a supine position on a measuring table or a floor mat with arms adjacent to, but not touching the body, palms flat against the table, and legs adjacent to each other but not touching. Four surface, self‐adhesive spot electrodes were placed on the dorsal surface of the right hand and on the dorsal surface of the right foot. Prior to electrode placement, the skin was cleansed with alcohol at the four locations for electrode placement. Resistance (R) and reactance (Xc) values were determined on the right side of the body. After the electrodes had been positioned, two readings were recorded. The mean of two readings for R and Xc was used in the analysis. Resistance index (RI = HT^2^/R) was calculated from HT and R.^28^ Waist‐to‐hip ratio (WHR) was calculated by dividing the waist circumference by hip circumference. Waist‐to‐height ratio (WHtR) was calculated by dividing the waist circumference by height.

### 2.4 Measurement of arterial stiffness

Measures of arterial stiffness were determined at baseline and in response to AngII infusion, as outlined above. Peripheral arterial pressure waveforms were recording by application tonometry using Sphygmocor system. Pulse‐wave velocity (PWV) was determined by sequential acquisition of pressure waveform from two sites sequentially (carotid and femoral arteries). The timing of the waveforms was compared with that of the R‐wave on a simultaneously recorded ECG. The distance travelled was calculated by subtracting the distance from carotid pulsation to femoral pulsation, following the anatomic line to avoid the influence of breast and abdomen on the distance measurement. The aortic augmentation index (AIx) was determined by applanation tonometry of the right radial artery using a Millar piezoresistive pressure transducer (Millar SPT 301; Millar Instruments, Houston, TX) coupled to a Sphygmocor device (PWV Medical).

### 2.5 Analysis

Clinical and biochemical characteristics are reported as mean ± SE for continuous variables and as total number followed by percentages for dichotomous variables. Sex differences of continuous, normally distributed variables were assessed using t tests. The primary analysis in this exploratory study was to determine the association between BMI and arterial stiffness at baseline and in response to Ang II infusion after 60 min, as a marker of arterial RAAS activity, stratified by sex. The secondary analysis was to determine the association between all other measures of adiposity and arterial stiffness at baseline and in response to Ang II challenge stratified by sex. All associations were evaluated using linear regression analysis. Associations between measures of adiposity and arterial stiffness, at baseline and in response to Ang II challenge, were analysed by univariate and multivariate linear regression. The following variables were included in multivariate analysis: age, mean arterial pressure (MAP). Baseline measures of arterial stiffness (baseline PWV or AIx) were also included in the analysis of their respective responses to AngII infusion.^29^ A sensitivity analysis excluding participants ≥30 years did not significantly alter the results. All model assumptions were tested and met. All statistical analyses were performed with the statistical software STATA and were two‐tailed with a significance level of 0.05. We a priori elected to compare the associations between measures of adiposity and the arterial responses to Ang II infusion as a marker for local RAAS activity, between females and males.^25^

## 3 RESULTS

### 3.1 Baseline characteristics

Baseline characteristics are shown in Table 1. The majority of participants self‐identified as white and approximately two‐thirds were female. Females had lower measures of blood pressure compared to males (systolic *p *= .04; diastolic, *p *= .05), and all participants were normotensive, non‐diabetic and in high‐salt balance, a state of maximal RAAS suppression. Females were shorter (*p *= .05) and weighed less (*p *= .05) than males but no sex differences were observed in BMI (*p *= .7). Waist circumference (*p *= .03) and WHR (*p <* .05) measures were lower and FM percentage higher (*p *< .001) in females compared to males (*p *= .03) but were within sex‐specific normal range.^30^ Hip circumference (*p *= .45) and WHtR (*p *= .61) did not differ by sex. Plasma renin activity (*p *< .001) and aldosterone (*p *< .001) levels were lower in females; Ang II levels did not differ by sex.

**TABLE 1:** Subject baseline characteristics

	Female (*n* = 67)	Male (*n* = 28)
Age (y)	37 ± 1.6	39 ± 2.6
Race (% white)	75	84
BMI (kg/m2)	25.3 ± 0.5	26.1 ± 0.7
HbA1c	5.4 ± 0.1	5.4 ± 0.1
SBP (mmHg)	111 ± 1.5	120 ± 3^a^
DBP (mmHg)	66 ± 1.1	70.1 ± 1.7^a^
MAP (mmHg)	81 ± 1.1	87 ± 2^a^
Ang II (ng/L)	19.5 ± 1.7	19.3 ± 1.5
PRA (ngAngI/ml per h)	0.19 ± 0.02	0.34 ± 0.04^a^
Aldosterone (pmol/L)	118 ± 9	166 ± 22^a^
WC (cm)	82.5 ± 1.4	92.2 ± 1.8^a^
HC (cm)	100.2 ± 1	100 ± 1.3
WHR	0.82	0.92^a^
WHtR	0.5	0.51
FM (%)	32.8 ± 1	21.1 ± 3^a^
PWV (m/s)	7.2 ± 0.2	8.1 ± 0.3^a^
AIx (%)	11.8 ± 1.7	2.9 ± 3.2^a^

Note

Values are mean ± SE.

Abbreviations: BMI, body mass index; DBP, diastolic blood pressure; FM: fat mass; HC, hip circumference; MAP, Mean arterial pressure; SBP, systolic blood pressure; WC, waist circumference; WHR, waist‐to‐hip ratio; WHtR, waist‐to‐height ratio.

^a^ *p* < .05 for differences vs. Females.

### 3.2 Baseline measures of arterial stiffness

As anticipated, females demonstrated lower baseline PWV (*p *< .01) but higher AIx values (*p *< .01) compared to males; however, all values for both sexes were within normal range.^31^ No association was observed between any measures of adiposity and baseline PWV by either uni‐ or multi‐variate analysis (Table 2) in female participants. In contrast, BMI, WC, WHtR and FM% were all positively associated with baseline PWV by both univariate and multi‐variate analysis in male participants (Table 3).

**TABLE 2:** Baseline univariate and multivariate analysis of the association between measures of adiposity and arterial stiffness in females

	PWV		AIx	
	Univariate r^2^	Multivariate r^2^	Univariate r^2^	Multivariate r^2^
BMI	0.0022	0.29	0.10^a^	0.60^a^
WC	0.0022	0.31	0.12^a^	0.61^a^
HC	0.0027	0.34	0.055	0.63^a^
WHR	0.04	0.30	0.14	0.65^a^
WHtR	0.005	0.24	0.12^a^	0.64^a^
FM	0.018	0.32	0.16^a^	0.62^a^

Abbreviations: AIx, augmentation index; BMI, body mass index; FM: fat mass; HC, hip circumference; PWV, pulse wave velocity; WC, waist circumference; WHR, waist‐to‐hip ratio; WHtR, waist‐to‐height ratio.

^a^ *p *< .05 for significant correlation.

^b^ *p*< .05 for significant interaction term with sex.

**TABLE 3:** Univariate and multivariate analysis of the association between measures of adiposity and arterial stiffness in males

	PWV		AIx	
	Univariate r^2^	Multivariate r^2^	Univariate r^2^	Multivariate r^2^
BMI	0.10^a^	0.4^a^	0.01	0.45^a^
WC	0.03^a^	0.33^a^	0.05	0.47^a^
HC	0.002	0.27	0.01	0.43^a^
WHR	0.02	0.25	0.02	0.45^a^
WHtR	0.11^a^	0.30^a^	0.15^a^	0.48^a^
FM	0.04^a^	0.39^a^	0.001	0.40^a^

Abbreviations: AIx, augmentation index; BMI, body mass index; FM: fat mass; HC, hip circumference; PWV, pulse wave velocity; WC, waist circumference; WHR, waist‐to‐hip ratio; WHtR, waist‐to‐height ratio.

^a^ *p* ≤ .05 for significant correlation.

^b^ *p* ≤ .05 for significant interaction term with sex.

Conversely, BMI, WC, WHtR and FM were positively associated with baseline AIx on both uni‐ and multivariate analysis in female participants. In male participants, WHtR was the only measure of adiposity associated with baseline AIx by univariate analysis, though after adjustment for age and mean arterial pressure, all measures of adiposity were significantly associated with baseline AIx.

### 3.3 Arterial stiffness response to angiotensin II challenge

**FIGURE 1:**
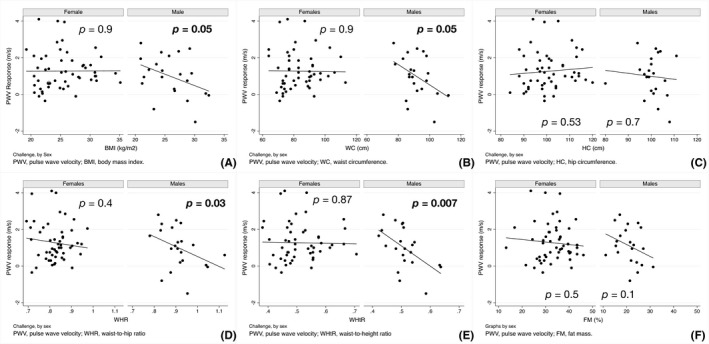
PWV response to angiotensin II challenge as a function of adiposity measures, by sex

**FIGURE 2:**
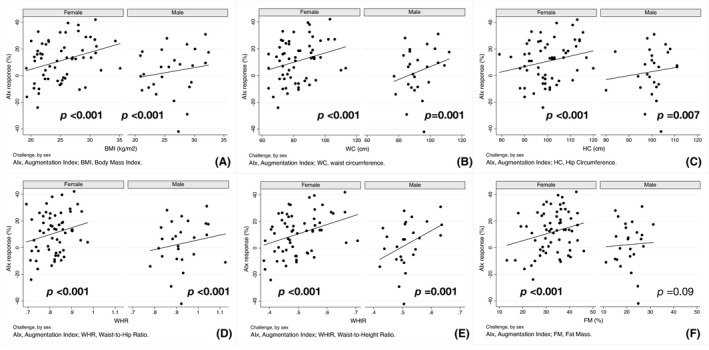
AIx response to angiotensin II challenge as a function of adiposity measures, by sex

No association between any measure of adiposity and the PWV response to AngII challenge was observed in female participants (Figure 1). In contrast, BMI, WC, WHR and WHtR were each significantly associated with the PWV response to AngII challenge in male participants (Figure 1). Conversely, all measures of adiposity were significantly associated with AIx in response to Ang II challenge in both female and male participants, though the associations did not differ by sex (Figure 2). No significant interaction was observed between sex and any of the arterial responses to Ang II challenge.

## 4 DISCUSSION

This exploratory study aimed to investigate sex‐specific associations between measures of adiposity and arterial stiffness and RAAS activity, validated markers of cardiovascular risk,^32^, ^33^, ^34^ in a young adult population across a wide range of BMI measures. Our key findings were (1) in female participants, no measure of adiposity was associated with PWV either at baseline or in response to AngII challenge; in contrast, most (including BMI) were significantly associated with both of these outcomes in male participants; (2) all measures of adiposity were associated with AIx, both at baseline and in response to AngII in both female and male participants. This suggests that while increased adiposity is a risk factor for cardiovascular disease^,^,^1^, ^4^, ^9^, ^13^, ^35^ how it affects cardiovascular risk appears to differ in young adult females compared to males, highlighting the need for sex‐specific standards for measures of adiposity to determine risk profile.

Differences in fat composition by sex are well‐recognized, with females having a greater body fat percentage than males.^16^, ^36^, ^37^ Although BMI is the most widely used clinical tool to measure adiposity,^1^ fat distribution is a stronger predictor of metabolic health.^19^, ^36^ Female adiposity is characterized by approximately 10% higher percent body fat with relatively more adipose tissue in the hips and thighs.^17^, ^38^ This female‐pattern fat distribution, independent of total body fat, has been associated with lower risk of type 2 diabetes and atherosclerosis.^16^, ^17^, ^18^, ^39^, ^40^ In contrast, male‐pattern adiposity is characterized by fat accumulation around the abdomen, which is associated with increased cardiovascular risk.^41^, ^42^, ^43^

Adipose tissue is the second most abundant source of local RAAS components.^44^ Studies show that visceral adipocytes express higher angiotensinogen (AGT) mRNA compared to subcutaneous adipose depots^45^, ^46^; which may be associated with the increased cardiovascular risk via activation of the RAAS.^47^ Schorr et al. investigated 208 overweight/obese subjects of similar age and BMI and reported lower visceral adipose tissue in females compared to males.^18^ Angiotensin II, angiotensin‐converting enzyme and AT_1_R are expressed in white adipose tissue, with higher expression in visceral versus subcutaneous depots.^48^ While premenopausal females have more adipose tissue, it is disproportionately brown adipose tissue^49^ which has lower levels of AT1R and AT2R expression and thus lower RAAS activation.^50^

Pulse wave velocity measures stiffness of an arterial segment. AIx is defined as the increment in pressure after the first systolic shoulder to the peak of the aortic pressure expressed as a percentage of aortic pulse pressure, and is higher in females than males.^51^ Previous work has highlighted that these separate measures of arterial stiffness and surrogate markers of cardiovascular risk are correlated, at least in healthy populations, and the strength of the correlation increases when participants are stratified by sex.^52^

Previous studies have suggested, however, that there is differential control of PWV and AIx. In a study of participants with cardiac disease,^53^ AIx was changed by the vasodilator nitroglycerin but PWV was not. In a study of healthy participants,^54^ AIx was significantly decreased during beta‐adrenergic stimulation, but PWV remained unchanged. While these studies did not stratify their results by sex, previous studies have shown that nitric oxide (NO) production is higher in the systemic vasculature of females than males. Female hearts have a decreased capacity to respond to β‐adrenergic stimulation,^55^ though no sex differences were observed in ovariectomized mice.^56^ In women, the rate of 8‐bromo‐cAMP‐ and isoprenaline‐stimulated lipolysis was approximately twofold and 1.5‐fold higher, respectively, in subcutaneous compared to omental adipocytes, whereas there was no difference between the two depots in men,^57^ suggesting that both sex and fat distribution play a role in modulating the relationship between beta‐adrenergic responsiveness and arterial stiffness. Estradiol is a potent stimulus for formation of endothelial NO synthase and subsequent generation of NO.^58^ The co‐presence of oestrogen receptors and β‐adrenergic receptors in endothelial cells of both sexes suggests their possible implication in sex‐specific cardiovascular regulation.^59^ All female participants in our study were premenopausal, suggesting that estradiol may have played a role in our findings, though this remains speculative.

Concerns have been previously raised regarding the underrepresentation of women in clinical studies.^60^, ^61^ Our study highlights the potential problems associated with generalizing findings to disparate populations. Oestrogens, directly or through activation of their receptors on adipocytes, support α2‐adrenergic receptors in subcutaneous adipocytes but have no effect on adrenergic receptors in visceral adipocytes.^62^ This may explain the sex differences in baseline PWV we observed between young female and male participants, and specifically the lack of association between any measure of adiposity and arterial stiffness as measured by PWV in young women.

This study has strengths and limitations. First, more females than males were included in the study; however, we ensured that our recruitment strategy and advertising was gender neutral to minimize any potential bias. Female and male participants were of similar age, and all female participants were premenopausal. Next, there is variation of the RAAS throughout the menstrual cycle,^15^, ^63^, ^64^ and use of oral contraceptives is associated with upregulation of RAAS activity.^65^, ^66^, ^67^, ^68^ However, female participants were studied during the same phase of the menstrual cycle, and those on hormonal contraception were excluded. Third, salt intake is a major determinant of RAAS activity, and as such, we ensured that all participants were in high‐salt balance, a state of maximum RAAS suppression.^27^ While the cross‐sectional study design limits the ability to draw conclusions about causality, the goal of the study was to determine whether the association between BMI and other measures of adiposity, arterial stiffness and RAAS activity differed by sex. By studying healthy fasting participants at the same time of day in a controlled setting with the same individual measuring arterial stiffness, we reduced the presence of confounders. There were sex differences in the baseline blood pressure and waist circumference measurements. We aimed to employ a sex‐informed methodology to increase the rigour and expand the relevance of this study to a broad population.^69^ Sex differences in normal blood pressure and fat distribution have been previously well‐described, with healthy females having lower blood pressure, mean arterial pressure and waist circumference than males.^69^, ^70^ Similarly, the World Health Organization has highlighted a lower cardiovascular disease risk with different waist circumference thresholds (<88 cm for women, <102 cm for men).^71^ The purpose of the study was to determine the associations between measures of adiposity and arterial stiffness, a validated marker of cardiovascular risk, in a healthy population. As the incidence of cardiovascular disease proportionately increased beginning at a lower range of SBP and waist circumference in women compared with men, participants with measures in the lowest sex‐specific cardiovascular risk ranges were enrolled.^72^ Lastly, our study population was young, self‐identified primarily as white and healthy, which may limit the generalizability of our findings. Arterial stiffness is directly correlated with age^73^ and ethnic differences in adiposity distribution are well‐recognized.^74^

In conclusion, our results suggest sex differences exist between measures of adiposity measures and cardiovascular risk as measured by arterial stiffness and arterial RAAS activity. These findings add to the growing literature of sex‐ and age‐specific assessment of cardiovascular risk. Future research is needed on sex‐specific tools that include measurements of adiposity along with other common clinical measures of cardiovascular risk.

**Perspectives and Significance**

Sex differences in human body composition are well‐recognized, with females generally having a greater body fat percentage. Although BMI is the most widely used clinical tool to measure obesity, it does not differentiate between type of adiposity or fat distribution, which both differ by sex. Our study suggests that while increased adiposity is a risk factor for cardiovascular disease, the mechanisms underlying the association of adiposity with cardiovascular risk differ in young adult females compared to males, highlighting the need for sex‐specific standards for measures of adiposity to determine cardiovascular risk.

## CONFLICT OF INTEREST

All authors have no conflicts of interest to disclose.

## AUTHOR CONTRIBUTIONS

**Cindy Kalenga:** Conceptualization (lead); Data curation (equal); Formal analysis (lead); Investigation (equal); Methodology (equal); Project administration (equal); Writing – original draft (equal). **Sharanya Ramesh:** Conceptualization (equal); Data curation (equal); Investigation (equal); Methodology (equal); Project administration (equal); Writing – review & editing (equal). **Sandra Dumanski:** Conceptualization (equal); Data curation (equal); Investigation (equal); Methodology (equal); Writing – review & editing (equal). **Jennifer MacRae:** Conceptualization (equal); Data curation (equal); Methodology (equal); Supervision (equal); Writing – review & editing (equal). **Kara Nerenberg:** Conceptualization (equal); Formal analysis (equal); Methodology (equal); Supervision (equal); Writing – review & editing (equal). **Amy Metcalfe:** Formal analysis (equal); Methodology (equal); Resources (equal); Supervision (equal); Writing – review & editing (equal). **Darlene Sola:** Data curation (equal); Investigation (equal); Methodology (equal); Project administration (equal); Writing – review & editing (equal). **Sofia B Ahmed:** Conceptualization (lead); Data curation (lead); Formal analysis (equal); Funding acquisition (lead); Investigation (equal); Methodology (equal); Project administration (equal); Resources (equal); Software (equal); Supervision (lead); Writing – original draft (lead); Writing – review & editing (equal).

## ETHICAL APPROVAL AND CONSENT TO PARTICIPATE

The study protocol was approved by the Conjoint Health Research Ethics Board at the University of Calgary. Written informed consent was obtained from all study participants in accordance with the Declaration of Helsinki.

## CONSENT FOR PUBLICATION

Not applicable.

## Data Availability

The datasets used and analysed during the current study are available from the corresponding author on reasonable request.
